# Exploration for olive fruit fly parasitoids across Africa reveals regional distributions and dominance of closely associated parasitoids

**DOI:** 10.1038/s41598-021-85253-y

**Published:** 2021-03-17

**Authors:** Xingeng Wang, Vaughn M. Walton, Kim A. Hoelmer, Charles H. Pickett, Arnaud Blanchet, Robert K. Straser, Alan A. Kirk, Kent M. Daane

**Affiliations:** 1 Department of Environmental Science, Policy and Management, University of California, Berkeley, CA 94720 USA; 2 USDA-ARS, European Biological Control Laboratory, Montferrier, France; 3 California Department of Food and Agriculture, Sacramento, CA 95832 USA; 4 Present Address: USDA-ARS Beneficial Insects Introduction Research Unit, Newark, DE 19713 USA; 5 Present Address: Department of Horticulture, Oregon State University, Corvallis, OR 97331 USA; 6 Present Address: Department of Entomology, University of California, Riverside, Riverside, CA 92521 USA

**Keywords:** Ecosystem services, Invasive species

## Abstract

The olive fruit fly, *Bactrocera oleae*, has been a key pest of olives in Europe and North America. We conducted the largest exploration for parasitoids associated with the fly across Sub-Saharan Africa (Kenya, Namibia, and South Africa) including some of the fly’s adjoining regions (Canary Islands, Morocco, Réunion Island and Tunisia). From Sub-Saharan regions, four braconids were collected: *Bracon celer*, *Psytallia humilis, P. lounsburyi*, and *Utetes africanus*. Results showed that their regional dominance was related to climate niches, with *P. humilis* dominant in hot semi-arid areas of Namibia, *P. lounsburyi* dominant in more tropical areas of Kenya, and *U. africanus* prevalent in Mediterranean climates of South Africa. *Psytallia concolor* was found in the Canary Islands, Morocco and Tunisian, and the Afrotropical braconid *Diachasmimorpha* sp. near *fullawayi* on Réunion Island. Furthermore, we monitored the seasonal dynamics of the fly and parasitoids in Cape Province of South Africa. Results showed that fruit maturity, seasonal variations in climates and interspecific interactions shape the local parasitoid diversity that contribute to the low fly populations. The results are discussed with regard to ecological adaptations of closely associated parasitoids, and how their adaptations impact biocontrol.

## Introduction

Exotic insect pests often thrive in their invaded regions due to the absence of specialist natural enemies and lack of effective indigenous natural enemies^1,2^. Classical biological control (CBC) by the introduction of specialist natural enemies from the exotic pest’s native range is an attempt to restore the pest-natural enemy balance after an invasion event^3–5^. Economic returns on successful programs are overwhelmingly positive^6^, but CBC programs require proper steps to be successfully implemented to minimize the number of natural enemy releases that are inconsequential or have negative non-target impacts on recipient ecosystems^7,8^. For herbivorous invaders, this requires a fundamental understanding of the natural enemy impact in its native range, biology and host specificity, as well as potential tri-trophic interactions that develop from a high degree of co-adaptation between plant—phytophagous pest—entomophagous arthropods and the impact of habitat and environment on the selected natural enemy^7^.

One aspect is matching the climatic niches occupied by the natural enemies in the native range to the invaded range^9^. Climate matching has been particularly important in fruit fly biological control programs^10,11^. Hymenopteran parasitoids from the braconid subfamily Opiinae have been used worldwide in CBC programs to control fruit-infesting Tephritidae^12–16^. The vast majority of utilized braconid parasitoids are koinobiont endoparasitoids that oviposit in the host egg or larval stage and emerge from host pupae^17^. Therefore, the adult female parasitoid must first locate and attack the concealed immature stages of host fly inside the fruit, then bypass the host immune response, and successfully develop. For these reasons, opiine parasitoids are generally well adapted to their associated host species.

The olive fruit fly, *Bactrocera oleae* (Rossi) (Diptera: Tephritidae) has been a key pest of cultivated olives throughout the Mediterranean Basin and North America, largely due to a lack of effective natural enemies in these invaded regions^18^. The fly larvae feed exclusively in olives^19^, both cultivated olives, *Olea europaea* ssp. *europaea* (Wall ex G. Don), and wild olives, of which various subspecies occur widely in parts of Africa, southern Europe and southwestern Asia^20,21^. The fly’s current range extends throughout the Mediterranean Basin, northern and Sub-Saharan Africa, southwestern Asia (parts of India, Pakistan and China), and North America (California and Mexico)^18,21^. Population structure and genetic analyses suggest that *B. oleae* is native to Sub-Saharan Africa and then likely moved into North Africa and later the Mediterranean Basin, westward through Europe into Asia, and eventually into North America^22–24^. The close association of the fly and olives suggests the existence of highly specialized parasitoids associated with *B. oleae*. In fact, indigenous parasitoids attacking *B. oleae* in the Mediterranean Basin are generalist chalcidoids such as *Eurytoma martellii* Dom. (Eurytomidae), *Eupelmus urozonus* Dalm. (Eupelmidae), *Pnigalio mediterraneus* Ferrière & Delucchi (Eulophidae) and *Cyrtoptyx latipes* Rond. (Pteromalidae)^25–27^. *Eupelmus urozonus* is a species complex, and several species from this complex have been recorded from *B. oleae* in Europe^28^. Similarly, the indigenous parasitoid attacking *B. oleae* in California, *Pteromalus* nr. sp. *myopitae* (Pteromalidae), is also a generalist^29^. These species are idiobiont ectoparasitoids, placing their eggs on the host surface and not needing to overcome internal host defenses, thus are polyphagous, attacking even unrelated insect hosts. While present, these generalist parasitoids do not currently provide effective *B. oleae* control.

The first major attempt to introduce coevolved parasitoids to suppress *B. oleae* populations dates to the early 1900s with the exploration for natural enemies in Africa to be released in Italy by Filippo Silvestri^30^. These early explorations discovered and described several braconid species collected from *B. oleae* including *Bracon celer* Szépligeti, *Psytallia concolor* (Szépligeti), *P. lounsburyi* (Silvestri) and *Utetes africanus* (Szépligeti) collected in South Africa, Kenya and Ethiopia, reviewed in^18,31,32^. However, none of these parasitoids were successfully cultured by Silvestri and only small numbers were released in Italy without subsequent establishment^33^. Only *P. concolor*, obtained from Tunisia, has been repeatedly introduced to the Mediterranean Basin since the early 1900s, but this species has established only in some southern regions and does not provide effective control^34,35^. Still, there has been continued interest in mass-rearing and releasing *P. concolor* and/or *P. lounsburyi* to improve sustainable fly management in Europe^36–39^.

The invasion and widespread establishment of *B. oleae* in California and northwestern Mexico initiated renewed interest in the classical biological control of this pest^40^. Modern exploration for effective natural enemies was designed to include the fly’s likely native ranges in Sub-Saharan Africa (Kenya, South Africa and Namibia) and some expanded regions in Africa (Canary Islands, Morocco, Tunisia and Réunion Island). Here we present a comprehensive analysis of the regional distribution, diversity, and relative dominance of braconid *B. oleae* parasitoids in these seven African regions and examine how the regional dominance might be associated with regional climatic variables. Furthermore, we analyzed the seasonal dynamics of *B. oleae* and its associated parasitoids in South Africa, one of the native regions with a high diversity of host-specific parasitoids and examined how some biotic and abiotic factors might have shaped the local diversity of the parasitoid complex that contribute to maintaining *B. oleae* populations at low levels. This framework may provide new insights into the nature of climate niches of different parasitoid species and their associated tri-trophic interactions in guiding ongoing biological control programs in California and the Mediterranean Basin.

## Results

### Parasitoid regional distribution and diversity

**Figure 1:**
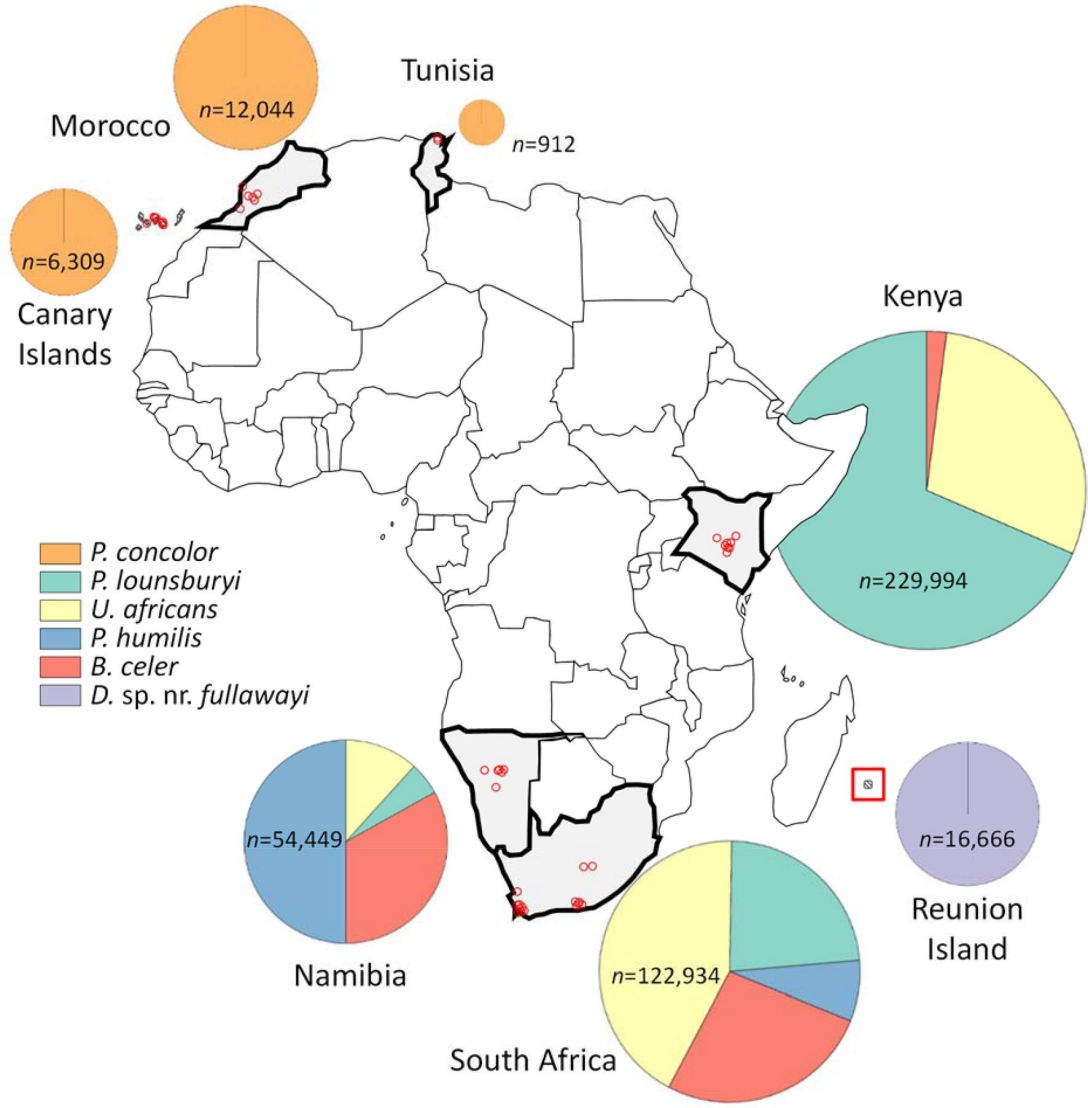
Composition and relative abundance (%) of braconid parasitoid species reared from *Bactrocera* spp. on wild olives in seven regions of Africa; small red circles show approximate locations of sampling sites and the parasitoid composition size is proportional to the number of fruits (*n*) collected in each region. The map was created in R (version 3.6.3) using ‘rworldmap’ package (version 1.3.6, https://cran.r-project.org/web/packages/rworldmap/rworldmap.pdf) ^69^.

Surveys from 110 sites of wild olives, *O. europea* nr. ssp. *cuspidata*, in seven African regions yielded a total of 443,308 olive berries (Fig. 1), from which 72,453 fly pupae, 27,848 adult *B. oleae* and 22,576 adult braconid parasitoids were obtained (Table 1). Two closely related African *Bactrocera* species, *B. biguttula* (Bezzi) (1.2%) and *B. munroi* White (2.6%) were also recovered, but in low numbers and with *B. biguttula* found only in South Africa and *B. munroi* only in Kenya (Table 1). Five Opiinae braconid wasps were recovered: *Diachasmimorpha* sp. near *fullawayi*, *P. concolor*, *P. humilis* (Szépligeti), *P. lounsburyi* and *U. africanus*; one Braconinae braconid wasp, *B. celer*, was also recovered. *Psytallia concolor* was the only species found in the Canary Islands, Tunisia and Morocco, whereas *D.* sp. near *fullawayi* was the only species recovered in the Réunion Island (Fig. 1). The other four species were found in Namibia and South Africa and three of them (except *P. humilis*) were found in Kenya, with *P. lounsburyi*, *P. humilis* and *U. africanus* being the predominant parasitoid species in Kenya, Namibia and South Africa, respectively (Fig. 1).

**Table 1:** Numbers of wild olive fruit collected, fly pupae, adult flies of *B. oleae*, *B. biguttula* and *B. munroi,* and braconid parasitoids obtained from the collections in different regions during 2002 to 2011.

Region	Year (no. of sites)	Fruit	Fly pupae	*B. oleae*	*B. biguttula*	*B. munroi*	Braconids
Tunisia	2000 (3)	912	710	602	0	0	108
Morocco	2004 (7)	12,044	487	316	0	0	41
Réunion	2004 (8)	16,666	1786	700	0	0	114
Namibia	2004 (3)	5595	756	413	0	0	188
	2005 (1)	700	35	17	0	0	1
	2007 (3)	15,862	2090	1034	0	0	601
	2008 (6)	11,583	5440	1402	0	0	1700
	2009 (4)	7070	1539	884	0	0	218
	2011 (2)	13,639	11	6	0	0	1
Kenya	2002 (1)	45,762	10,364	3309	0	0	5039
	2003 (2)	71,680	16,291	5177	0	0	8432
	2004 (1)	440	100	38	0	0	21
	2005 (5)	41,008	9321	4020	0	335	2125
	2006 (1)	29,040	6600	1834	0	21	264
	2007 (3)	28,864	6560	2277	0	16	2402
	2008 (2)	13,200	3000	1314	0	108	668
South Africa	2003 (29)	74,579	2323	1095	0	0	244
	2004 (16)	25,885	1095	400	0	0	157
	2005 (6)	22,470	800	388	23	0	125

**Figure 2:**
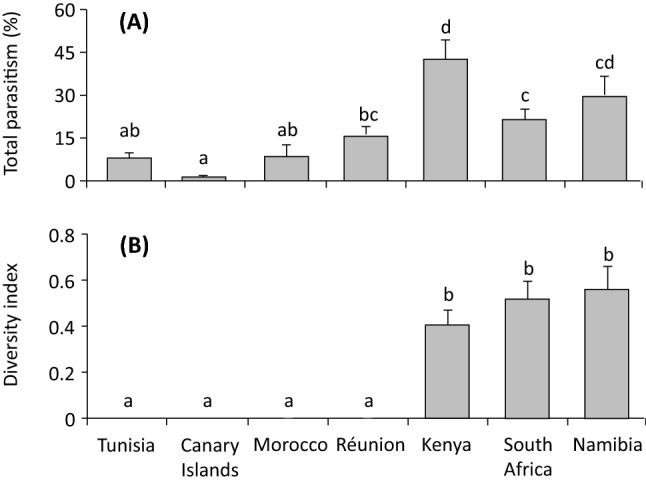
(**A**) Apparent percentage parasitism of *Bactrocera* spp. (emerged parasitoids/(emerged parasitoids + flies)) and (**B**) parasitoid species diversity (Shannon index) of the braconid parasitoid species reared from the collected flies in different regions in Africa; bars refer to mean and SE and different letters above the standard error bars indicate significant differences (*P* < 0.05). PCA statistics and graphs were performed using JMP Pro ver13 (SAS, Cary, NC).

Total apparent parasitism was higher in the three Sub-Saharan regions than in other regions (*F*_6,84_ = 6.8, *p* < 0.001) (Fig. 2A). Diversity of the braconid parasitoid complex was similar among the three Sub-Saharan regions (*F*_6,66_ = 4.5, *p* < 0.001) (Fig. 2B). The percentage of female wasps was 64.4 ± 10.3% (n = 11, number of sites) for *P. concolor*, 50.3 ± 4.0% (n = 42) for *P. lounsburyi*, 65.4 ± 6.2% (n = 28) for *P. humilis*, 60.3 ± 3.5% (n = 50) for *U. africanus,* 43.4 ± 8.6% (n = 9) for *D.* sp. near *fullawayi* and 24.8 ± 4.8% (n = 35) for *B. celer*. The sex ratios were similar among the five opiine parasitoids but was higher than that of the braconine parasitoid *B. celer* (*F*_5,170_ = 7.9, *p* < 0.001).

**Figure 3:**
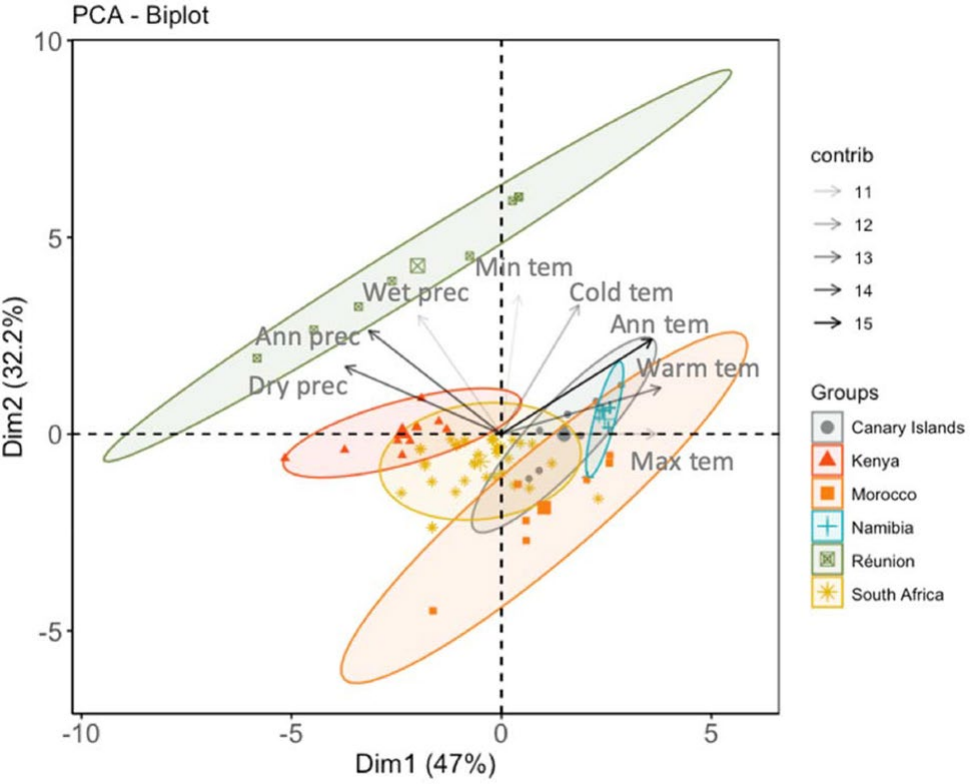
Principal Component Analysis (PCA) ordination of explored regions based on the two principal climatic gradients. The enlarged symbols indicate the centroids of the inertia ellipses while arrows indicate the importance of each bioclimatic variable on the two significant components. Climatic predictors are: *Ann tem* annual mean temperature, *Max tem* maximum temperature of the warmest month, *Min tem* minimum temperature of the coldest month, *Warm tem* mean temperature of the warmest quarter, *Cold tem* mean temperature of the coldest quarter, *Ann prec* annual precipitation, *Wet prec* precipitation of the wettest quarter, *Dry prec* precipitation of the driest quarter. The figure was created using JMP Pro ver13 (SAS, Cary, NC).

PCA revealed two major components that jointly explained 79.1% of the variance in the regional climatic variables (eigenvalues: component 1 = 3.76, 46.9% of variance; component 2 = 2.58, 32.2% of the variance) (Fig. 3). Despite the overlap of a few sites, the explored regions represented clearly different climate types. The climates in the Canary Islands, Morocco and Namibia were similar and are characterized by high temperatures and low precipitation. However, the climates in Kenya were related positively to the precipitation but negatively to the maximum temperature of the warmest month, with South Africa falling between these two climate types. The climates of Réunion Island are characterized by high precipitation and marked thermal contrasts across the island.

**Figure 4:**
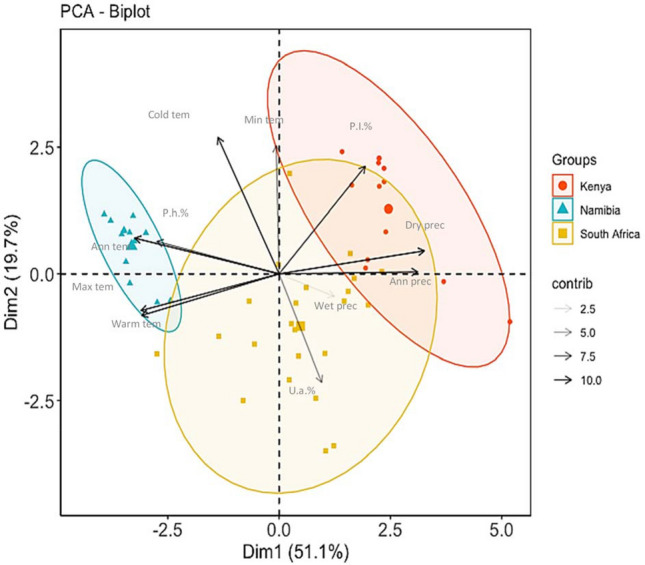
Principal Component Analysis (PCA) ordination of sampling sites in three Sub-Saharan countries based on the relative abundance of the dominant parasitoid species (*P. lounsburyi*, *P. humilis* and *U. africanus*). The biplot shows the relationships between bioclimatic variables and dominance of parasitoid species in each region. Climatic predictors are: *Ann tem* annual mean temperature, *Max tem* maximum temperature of the warmest month, *Min tem* minimum temperature of the coldest month, *Warm tem* mean temperature of the warmest quarter, *Cold tem* mean temperature of the coldest quarter, *Ann prec* annual precipitation, *Wet prec* precipitation of the wettest quarter, *Dry prec* precipitation of the driest quarter, and the relative proportion of *P. lounsburyi* (P.l.%), *P. humilis* (P.h.%) and *U. africanus* (U.t.%) to emerge from parasitized fruits. PCA statistics and graphs were performed using JMP Pro ver13 (SAS, Cary, NC).

The regional dominance of the parasitoid species in the three Sub-Saharan countries was reflected in the PCA ordination (eigenvalues: component 1 = 5.62, 51.1% of variance; component 2 = 2.17, 19.7% of the variance) (Fig. 4). Sites in Kenya were assigned on the left while sites in Namibia were assigned on the right, and those in South Africa were in the middle. There was a positive relationship between the annual mean temperature or maximum temperature of the warmest month and the relative abundance of *P. humilis*, however this relationship was negative for *P. lounsburyi*. The relative abundance of *U. africanus* was negatively correlated with the minimum temperature of the coldest month and strongly associated with the precipitation during the wettest quarter.

### Seasonal host-parasitoid dynamics

**Figure 5:**
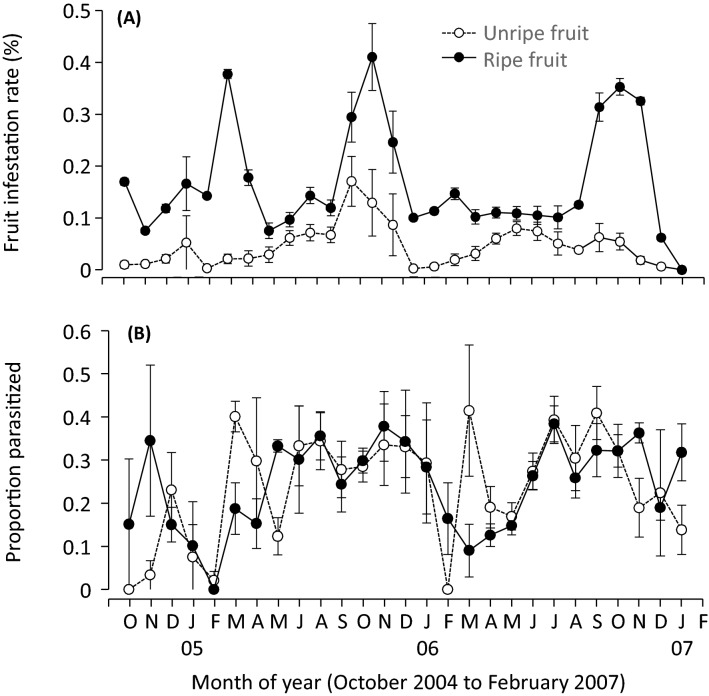
For both unripe and ripe fruit, the seasonal dynamics of (**A**) combined host density of *B. oleae* and *B. biguttula* and (**B**) apparent parasitism by braconid parasitoids from October 2004 to February 2007 in the Western Cape, South Africa. Values are mean and SE and data were pooled from different collection sites. The figure was created using JMP Pro ver13 (SAS, Cary, NC).

Independent of the regional surveys, more localized surveys were conducted at 24 sites of wild olives, *O. europaea* nr. ssp. *cuspidata*, in the Western Cape Province, South Africa, where a total of 252,603 unripe and 139,872 ripe wild olive fruit were collected (Table 2). Unripe fruit was significantly smaller (pulp thickness = 0.94 ± 0.04, n = 32) (*F*_1,73_ = 169.4, *p* < 0.001) than ripe fruit (mean ± SE pulp thickness = 1.93 ± 0.06, n = 43). Mean monthly host density (or fruit infestation rate) on unripe and ripe fruit were 4.5 ± 0.7% (range 0.5–11.7%) and 14.8 ± 2.0% (range 4.6–41.2%), respectively (Fig. 5A). Host density increased with fruit maturity and was affected by the interaction between fruit maturity and seasonal temperature (Table 3). Most emerged flies were *B. oleae*; only 0.87 ± 0.39% (mean ± SE) and 1.90 ± 0.66% (n = 24) of the emerged flies from the unripe and ripe fruit were *B. biguttula*. Combined parasitism of *B. oleae* and *B. biguttula* were 28.6 ± 2.8% (mean ± SE) and 25.4 ± 2.6% on the unripe and ripe fruit, respectively. Parasitism levels were not associated with fruit maturity but rather negatively related to mean temperature (Table 3), decreasing only during mid-summer months (Fig. 5B).

**Table 2:** Numbers of wild olives collected and fly pupae, adult flies of *B. oleae* (Bo) and *B. biguttula* (Bb) and braconid parasitoids emerged from collected unripe and ripe fruit in 26 different sites in the Western Cape, South Africa from October 2004 to February 2007.

Collection site (city, code)	Collections of ripe fruit					Collections of unripe fruit				
	Fruit	Pupae	Bo	Bb	Parasitoids	Fruit	Pupae	Bo	Bb	Parasitoids
Cape Town 1	552	63	29	0	5	208	13	5	0	6
Stellenbosch 2	14,338	3428	879	11	402	14,943	523	239	0	84
Stellenbosch 3	6927	949	275	17	119	9786	79	53	0	9
Stellenbosch 4	8002	1448	533	72	228	19,142	355	140	8	101
Stellenbosch 5	4795	1290	536	48	216	8825	287	111	9	55
Stellenbosch 6	11,162	1723	706	37	289	17,026	290	158	3	54
Stellenbosch 7	15,833	1176	453	8	192	32,580	382	168	3	66
Stellenbosch 8	919	78	67	0	5	891	51	35	0	3
Stellenbosch 9	3983	5	1	0	0	438	9	7	0	0
Stellenbosch 10	2073	50	27	0	11	4795	56	24	0	16
Bonnievale 1	5438	895	338	26	256	5965	248	85	3	83
Paarl 1	14,319	4530	2102	48	892	28,199	1151	632	5	259
Paarl 2	14,420	3425	1626	13	709	31,438	1387	613	0	377
Paarl 2	8706	1265	631	1	118	13,591	1031	612	1	119
Paarl 4	5665	694	360	0	154	16,166	549	267	0	154
Paarl 5	4342	288	157	2	57	9306	249	124	0	87
Paarl 6	7408	2040	856	2	350	11,062	1113	608	0	95
Wellington 1	7091	676	311	0	167	9923	198	94	0	58
Citrusdal 1	1323	124	54	0	13	2621	123	70	0	18
Citrusdal 2	2722	99	49	0	17	4595	78	31	0	20
Citrusdal 3	227	90	21	0	1	1335	193	76	0	26
Citrusdal 4	2052	114	55	0	28	3997	116	37	0	15
Citrusdal 5	222	24	4	0	6	515	32	13	0	8
Citrusdal 6	898	130	82	0	7	1711	230	174	0	8

**Table 3:** Mixed Models analyzing the effects of fruit maturity (unripe vs. ripe), mean monthly temperature as well as the interactions of these two factors on fruit infestation rate and apparent parasitism in Western Cape, South Africa.

Parameter	Variables	Estimate ± SE	*t*	*P*
% Fruit infested	Fruit maturity (FM)	0.098 ± 0.015	6.62	< 0.001
	Mean temperature (MT)	− 0.003 ± 0.003	0.97	0.335
	FM × MT	− 0.008 ± 0.003	2.24	0.029
Parasitism	Fruit maturity	− 0.039 ± 0.051	− 0.77	0.446
	Mean temperature	− 0.029 ± 0.012	− 2.47	0.017
	FM × MT	− 0.005 ± 0.012	− 0.40	0.688

**Figure 6:**
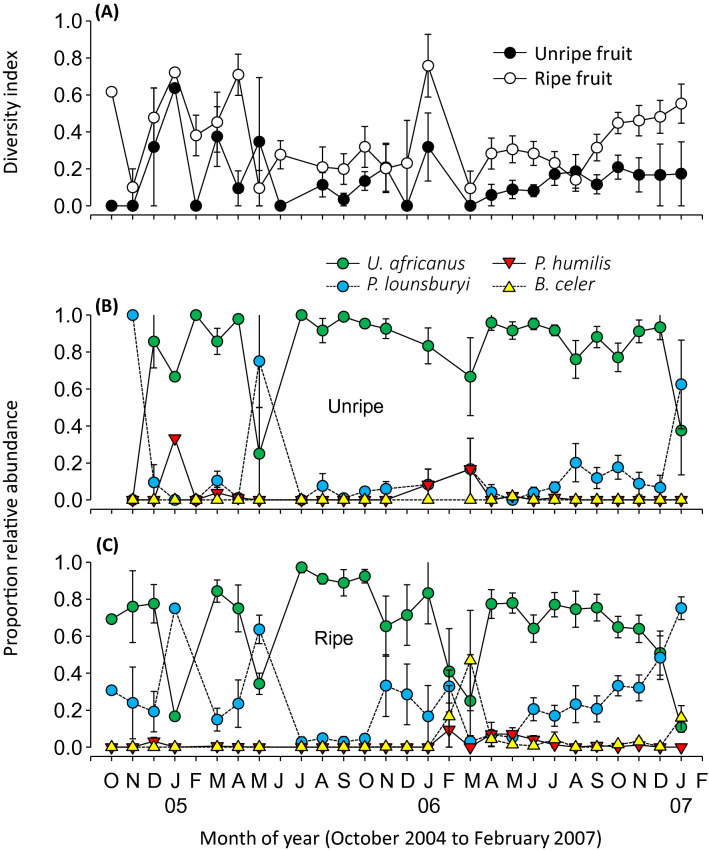
For both unripe and ripe fruit, the seasonal dynamics of (**A**) parasitoid species diversity (Shannon index) of the braconid parasitoid species reared from the collected flies, and relative abundance of braconid parasitoid species reared from *B. oleae* and *B. biguttula* on (**B**) unripe fruit and (**C**) ripe fruit in the Western Cape, South Africa. Values are mean and SE and data were pooled from different collection sites. The figure was created using JMP Pro ver13 (SAS, Cary, NC).

All four braconid parasitoids, *B. celer*, P. *humilis*, *P. lounsburyi* and *U. africanus,* were found in both unripe and ripe fruit. Diversity was generally lower in unripe than ripe fruit (Fig. 6A). *Utetes africanus* was the predominant parasitoid, followed by *P. lounsburyi* while both *P. humilis* (mean 1.8% and 2.5% on the unripe and ripe fruit, respectively) and *B. celer* (mean 0.1% and 4.9% on unripe and ripe fruit, respectively) were less common in both unripe (Fig. 6B) and ripe (Fig. 6C) fruit. GLM analyses showed that diversity was not affected by mean temperature but was positively related to fruit maturity and host density. The relative abundance of *U. africanus* was affected negatively by fruit maturity and the presence of other parasitoid species but was positively related to host density (Table 4). The relative abundance of *P. lounsburyi* was also affected positively by fruit maturity, seasonal temperature, and host density but negatively by the presence of other parasitoids (Table 4).

**Table 4:** Generalized Linear Model testing the effects of (1) fruit maturity (unripe vs. ripe), mean monthly temperature and host density (= % fruit infested) on diversity of braconid parasitoids, and (2) fruit maturity, mean monthly temperature, host density, and incidence of other braconids on the relative abundance of the dominant braconids *U. africanus* or *P. lounsburyi* in Western Cape, South Africa.

Parameter	Variables	Estimate ± SE	*Χ*^2^	*P*
Diversity	Fruit maturity	0.68 ± 0.23	9.70	0.002*
	Mean temperature	0.03 ± 0.02	1.92	0.166
	Host density	1.21 ± 0.43	7.05	0.008*
% *U. africanus*	Fruit maturity	− 0.64 ± 0.30	4.71	0.030*
	Mean temperature	− 0.04 ± 0.03	1.49	0.221
	Host density	2.17 ± 0.81	7.91	0.005*
	Presence of *P. lounsburyi*	− 2.63 ± 0.32	90.08	< 0.001*
	Presence of *P. humilis*	− 1.64 ± 0.40	16.88	< 0.001*
	Presence of *B. celer*	− 1.97 ± 0.53	15.11	< 0.001*
% *P. lounsburyi*	Fruit maturity	0.84 ± 0.32	7.52	0.006*
	Mean temperature	0.08 ± 0.03	4.78	0.029*
	Host density	1.75 ± 0.74	5.40	0.020*
	Presence of *U. africanus*	− 2.96 ± 0.43	50.48	< 0.001*
	Presence of *P. humilis*	− 1.29 ± 0.54	6.69	0.009*
	Presence of *B. celer*	− 0.18 ± 0.56	0.10	0.754

## Discussion

We conducted the largest modern exploration for olive fruit fly parasitoids in Africa. Our surveys reveal remarkable differences in distribution, diversity and dominance of braconid parasitoid guilds from wild olives across the African continent. The sub-Saharan regions of Namibia, South Africa and Kenya maintained the highest diversity of braconid *B. oleae* parasitoid species, supporting the argument of a Sub-Saharan origin of *B. oleae*^22–24^. We found only one native braconid parasitoid (*P. concolor*) in northern Africa, despite climates in the sampled regions being similar to that of Namibia. We recovered only *D.* sp. near *fullawayi* on Réunion Island, where the native Afrotropical species *Diachasmimorpha fullawayi* (Silvestri) was reported from other tephritid fruit flies^41^. No braconid parasitoids are reported to occur naturally in Europe’s Mediterranean Basin^25,26^ or California^40^. Although *P. concolor* was found in Corsica, France, where this parasitoid has never been released, it was uncertain if it was accidently introduced from established regions in Italy or North Africa^27^. Although not discussed here, surveys in Asia were also conducted in India, Nepal, Pakistan and China, and from these collections another braconid parasitoid, *Psytallia ponerophaga* (Silvestri), was reared from *B. oleae* in Pakistan and *D. longicaudata* (Ashmead) was recovered in China^21,42^.

All five parasitoids reared from *B. oleae* are larval parasitoids and four of them (*D.* sp. near *fullawayi*, *P. humilis*, *P. lounsburyi* and *U. africanus*) are koinobiont endoparasitic opiine wasps; *B. celer* is an idiobiont ectoparasitic braconine wasp^43–45^. Among tephritid fruit fly parasitoids, only a few are braconine parasitoids and nearly all of these are idiobiont ectoparasitoids of the larval flies^17^. No egg parasitoids of *B. oleae* were found in the current survey reported herein, or in previous surveys^12,30,32,33,46^, although one generalist egg parasitoid, *Fopius arisanus* (Sonan) (Hymenoptera: Braconidae), was able to attack and develop from *B. oleae* under quarantine conditions^47^. In our collections, parasitoids were obtained from pupae collected after exiting fruit or by rearing adults from infested fruit. It is likely that parasitoids that locate and attack hosts in the soil after larvae drop from fruit, or following pupation, have been underrepresented^46^. Some pupal parasitoids such as *Pachycrepoideus vindemiae* Rondani (Hymenoptera: Pteromalidae) were known to attack *B. oleae*^30^. Other chalcidoid parasitoid species were reported previously from Africa attacking fruit flies in multiple families, and they are considered to be relative generalists and would not be recommended for introduction for biological control e.g.,^48,49^. The other two closely related fly species recovered, *B. biguttula* in South Africa and *B. munroi* in Kenya, were also collected from wild olives. The collected parasitoids may also attack these two fly hosts, but *B. oleae* is thought to be their major host species as the number of the other two fly species were extremely low in South Africa and Kenya and not recovered in Namibia during our collections.

Four parasitoid species, *B. celer, P. humilis*, *P. lounsburyi* and *U. africanus*, were sympatric in the sub-Saharan regions surveyed. However, their dominance varied among regions with different climate types, as determined by PCA. In central Kenya where *P. lounsburyi* was the dominant species, the climate is characterized by mild tropical weather with relatively limited fluctuations in temperature extremes but ample precipitation during the rain months. In contrast, in Namibia where *P. humilis* was the dominant species the climate is typically hot and dry during summer and cold and humid during the winter. Indeed, laboratory studies confirmed that *P. humilis* was more heat-tolerant, yet less cold tolerant, than *P. lounsburyi*^11,50^, which may impact their establishment in regions with either hotter summers or colder winters. Although little is known about *U. africanus*’ temperature tolerance, the current surveys showed *U. africanus* was more abundant in the Mediterranean-like climates. Many other biotic and abiotic factors could also affect the distribution of these parasitoids. Rainfall patterns would strongly influence the seasonal occurrence and abundance of fruit availability, and consequently the abundance of flies and their parasitoids. In drier habitats, the fruit is likely to be small and ripen slowly, offering little food for fly larvae. Annual precipitation was consistently highest in Kenya, as were olive fly populations and their parasitoids (Table 1). Interspecific competition may occur and coexistence between these species is likely facilitated by niche segregation through differentiation in biological or ecological traits. As shown in South Africa, *U. africanus* was more dominant on small and unripe fruit whereas *P. lounsburyi* was more dominant on ripe fruit. Large ripe fruit may limit the access of *U. africanus*, which has the shortest ovipositor among all five larval parasitoids^10^, but other parasitoids such as *P. lounsburyi* fill the host feeding niches. If interspecific competition shapes the parasitoid guilds, it likely would show a similar dominance across different regions. Thus, adaptation to abiotic conditions is likely a major force underpinning diversification and dominance of these species.

The four braconid parasitoids recovered in sub-Saharan regions (*B. celer, P. humilis*, *P. lounsburyi* and *U. africanus*) have been imported and evaluated in classical biological control of *B. oleae* in California^51^, whereas *D.* sp. near *fullawayi* was not evaluated. In addition, *D. longicaudata* was also found to readily attack *B. oleae*^10,43,52^, but it is a generalist parasitoid of tephritids^41^. Among the well-adapted African braconid parasitoids, the relatively shorter length of *U. africanus* ovipositors match with lower pulp thickness of wild olives. This parasitoid is ineffective on cultivated olives that has higher pulp thickness through breeding programs. This thicker pulp allows *B. oleae* fly larvae to move deeper into the olive pulp to escape attack from larval parasitoids that have short ovipositors^10,52^. *Bracon celer* is able to attack the Cape ivy fly, *Parafreutreta regalis* Munro (Tephritidae: Tephritinae), which itself was introduced from South Africa into California for the control of the invasive Cape Ivy weeds^43,53^. *Psytallia concolor* is also a common parasitoid of the Mediterranean fruit fly, *Ceratitis capitata* (Wiedemann) (Diptera: Tephritidae) in eastern Africa^12,41,54^. Although the current surveys did not find it on *B. oleae* in Kenya, it has been previously collected from coffee-infesting *C. capitata* in other parts of Kenya^54^. Genetic analysis showed clear separation of the North African populations from the Sub-Saharan populations and thereafter referred the Sub-Saharan *P. concolor* populations (often described as *P*. cf. *concolor*^54,55^) as *P. humilis*^56,57^. *Psytallia lounsburyi* has been reared only from *B. oleae*^41,54,58,59^ and is the most host-specialized parasitoid among all parasitoid candidates^60^. In the Mediterranean Basin, *P. concolor* is the only parasitoid that has been extensively studied e.g.,^61^ and widely released with partial establishment in the southern regions^33,62^.

The current study also showed that fruit maturity, seasonal variations in climates and interspecific competition likely shape seasonal host-parasitoid dynamics in South Africa. Collectively, the co-adaptation of parasitoids and hosts may have contributed to maintaining low host population densities in its native range where fruit infestation rate was generally less than 15%. In contrast, untreated olives can reach 100% infestation in California^63^. Although olive fruit fly larvae are not tolerated in fruit used for canning, 10–30% infested fruit can be tolerated in olives that are pressed for oil in California. In California, two *P. humilis* populations, originated from *B. oleae* on wild olives in Namibia and *C. capitata* on coffee in Kenya, were released without subsequent establishment^40,64–66^. However, two populations of *P. lounsburyi*, originating from Kenya and South Africa, were released and successfully stablished along the California coastal regions^40^. Low winter survival may contribute to the failure of establishment of *P. concolor* in northern Mediterranean Basin^62^ and *P. humilis* in California^11,67^. For this reason, *P. ponerophaga* from Pakistan is being considered for release in California as it was found to have higher rates of low temperature survival than *P. humilis*^68^. Other factors such as availability of olive fruit for the host-specific *B. oleae* and alternative hosts could also restrict the establishment of its specialized parasitoids in introduced regions. In South Africa, wild olives are widely available all year, and alternative hosts may also help parasitoid populations to survive periods when local *B. oleae* populations are sparse^31,54^.

In conclusion, this study reveals the diversity, geographical distributions and dominance of parasitoids closely associated with *B. oleae* in the fly’s native range and provides a better understanding of potential ecological factors that may affect the efficiency and establishment of candidate parasitoids as biological control agents. In particular, identifying the suitable climatic niche of these different parasitoid species and understanding their geographic predictability helps to determine the potential establishment in released habitats in the presence of biotic interactions is paramount for successful biological control of *B. oleae*. Alteration of fruit morphological traits (such as size) through domestication may modify the tri-trophic interactions in agricultural eco-systems, reducing the efficiency of the larval parasitoids with short ovipositors on cultivated olives^10,43,52^. Therefore, understanding both the ecological niche and the co-evolutionary history of the host and parasitoid is fundamentally important for effective classical biological control. The recent successful establishment of *P. lounsburyi* in California should evoke further investigation into the use of this species for classical biological control of *B. oleae* in other climatically similar regions^31,46,51^. The advances of modern rearing techniques for these exotic parasitoid species and their tephritid hosts should further facilitate the use of classical and augmentative biological control of *B. oleae*^37^.

## Materials and methods

### Regional exploration

African collections for *B. oleae* and its parasitoids were conducted from 2000–2011 in seven regions: parts of Kenya, Namibia, South Africa, the Canary Islands, Tunisia, Morocco and Réunion Island (Fig. 1; for detailed locations of collection sites see Fig. S1). Each year, fruits of wild olives, *O. europea* nr. ssp. *cuspidata*, were collected, typically during the fruit ripening season in late summer or fall from various habitats including roadsides, hillsides, along stream banks and in woodland landscapes. Sample size (total number of collected fruit) varied among regions, sites and collection dates depending on the availability of fruit. Kenyan surveys were conducted from 2002–2008 at 15 sites in the forests along the southwestern slopes of Mount Kenya in Central Kenya. These sites were located near both sides of the equator and ranged in elevation from 1918–2557 m. Namibian surveys were conducted from 2004–2011 at 18 sites in the Otjozondjupa Province that ranged in elevation from 1409–1557 m. South African surveys were conducted from 2001–2005 at lower elevations (< 500 m) in provinces of the Western Cape (29 sites), Eastern Cape (10 sites) and Gauteng (two sites). The 2001 and 2002 data are not reported fully herein because parasitoid specimens were not always identified to species. Surveys in northern Tunisia were conducted in 2000 at three sites. Surveys in the Canary Islands, Morocco and Réunion Island were conducted in 2004, with nine sites on the Canary Islands (four on Tenerife, two on Gran Canaria and three on La Gomera), seven sites in the South Province of Morocco and eight sites on the Réunion Island.

Collected fruits were kept at room temperature (20–23 °C) in collaborating laboratories or hotel rooms near collection sites. The fly larvae often pupate inside unripe fruit but will exit and pupate outside of ripe fruit (typically in the soil underneath the tree in situ). When available, the majority of collected fruit were ripe, this allowed an easier collection of the fly puparia emerging from fruit, although both unripe and ripe fruit were collected. Larval ectoparasitoids, such as *B. celer*, emerge directly from fruit, which were held for up to one month for maximum emergence of flies or parasitoids. When possible, the emerged pupae were returned with the collectors or sent by cooperators to the ARS European Biological Control Laboratory (EBCL), otherwise the material was held at collaborating laboratories for emergence of flies or parasitoids.

All emerged insects were identified to species and gender. All parasitoid species were initially identified and/or subsequently confirmed by Dr. Robert Wharton (retired, Texas A&M university). Keys of these fruit fly parasitoids are available at http://paroffit.org^17,38^. These parasitoid species were further confirmed through molecular analyses^21,53^. Most of these parasitoid species can be easily identified based on the morphological characteristics as described by^17^, except for *P. humilis* and *P. concolor*; these two species are morphologically indistinguishable^52–54^. However, genetic analysis showed a clear separation of the north African and sub-Saharan populations^53^. According to Rugman-Jones et al.^53^, *P. humilis* is the attributed name for all sub-Saharan populations while *P. concolor* is the attributed name for all north African populations. The relationship of these two species was also confirmed in other studies^44,45^. All vouchers were deposited in the following institutional collections: USDA-ARS EBCL, University of California Berkeley, University of California Riverside and Texas A & M University, where various specimens were identified.

### Seasonal host-parasitoid dynamics

**Figure 7:**
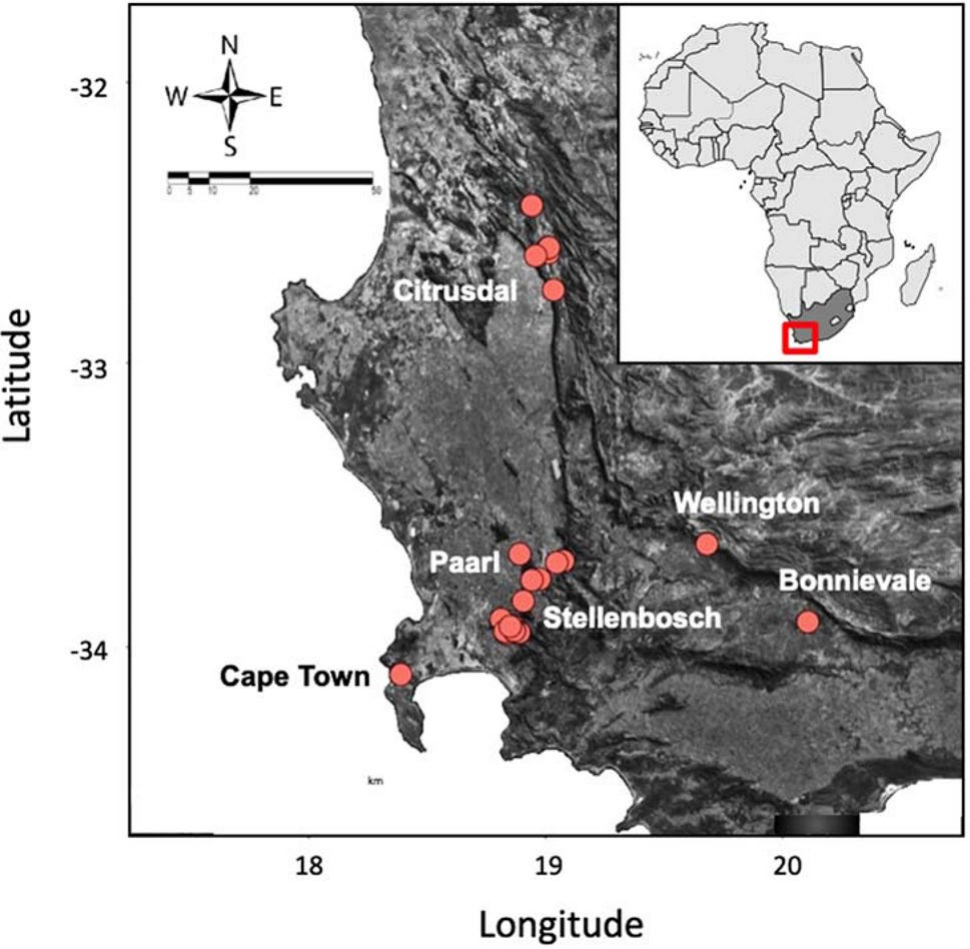
Sampled sites where the seasonal dynamics of *Bactrocera* spp. and their parasitoids were monitored (2004–2007) on wild olives in the Western Cape, South Africa. The map was created in R (version 3.6.3, www.r-project.org) using “get_map” wrapper from “ggmap” package (version 3.0.0, https://cran.r-project.org/web/packages/ggmap/ggmap.pdf), which queries with Google Maps server to produce static maps^69^.

To monitor the seasonal dynamics of *B. oleae* and its co-evolved parasitoids, collections of wild olives, *O. europea* ssp. *cuspidata*, were conducted from October 2004 to February 2007 at 24 fixed sites in the Western Cape Province, South Africa, near Bonnievale, Cape Town, Citrusdal, Paarl, Stellenbosch and Wellington (Fig. 7). These sites were located within 200 km of each other and ranged in elevation from 77–823 m. Approximately 900 fruits of wild olive were collected at each site once every 2–4 weeks, depending on the availability of fruit. Collected fruit were processed at Stellenbosch University and sorted by size and condition. Fruit size, or pulp thickness, was assumed to affect some parasitoid species ability to find and oviposit into fly larvae feeding deeper inside the fruit because of their short ovipositors^10,11^. Fully ripe (black) fruit is generally larger than unripe (green) fruit and fly larvae will feed deeper inside the softer fruit. Therefore, green and black fruit were sorted and assessed separately. Subsamples of unripe and ripe fruit were measured to estimate the pulp thickness of each fruit by inserting an insect pin trough the pulp to the seed three times at randomly selected points on the fruit. The mean depth (pin length minus the exposed portion of the pin) of the three measurements was used to estimate fruit pulp thickness. Collected fruit and emerging puparia were kept at room temperature (20–23 °C) until the emergence of wasps and flies.

### Data analysis

The relative abundance of each parasitoid species (i.e., percentage of each parasitoid species emerged), total parasitism by all parasitoids and diversity were estimated for each sample in each site and region. Total parasitism was calculated by dividing the total number of emerged parasitoids by the sum of the number of emerged parasitoids and flies (hereafter referred to as apparent parasitism, as the initial mortality was unknown). The Shannon index (*H*) was used to estimate the diversity:

$$H = \sum \left(pi\right)\left|\mathrm{ln}\left(pi\right)\right|$$

 where *pi* is the proportion of each parasitoid species. Sex ratio (% females) of each parasitoid species was pooled from different regions because initial analyses for each parasitoid species separately did not detect significant differences for any parasitoid among different regions (Table S1). Mean apparent parasitism and diversity among different regions and sex ratio among different parasitoid species were compared using one-way ANOVA. All data were first inspected for normality and error variance for homoscedasticity and all percentage data were logit transformed as needed before analysis.

Principal Component Analysis (PCA) was conducted to compare climate among the sampled regions (Tunisia was excluded due to the small samples and its climatic similarity to Morocco) and to analyze potential relationships among regional dominance of parasitoid species and bioclimatic variables in the three Sub-Saharan countries. A set of eight bioclimatic variables were selected for the analyses: annual mean temperature (Ann tem), maximum temperature of the warmest month (Max tem), minimum temperature of the coldest month (Min tem), mean temperature of the warmest quarter (Warm tem), mean temperature of the coldest quarter (Cold tem), annual precipitation (Ann prec), precipitation of the wettest quarter (Wet prec) and precipitation of the driest quarter (Dry prec). These bioclimatic variables were extracted from the WorldClim Global Climate Database 1.3 (http://www.worldclim.org) using the R 3.1.3 release. These variables are considered biologically relevant and used commonly in species distribution studies. A biplot analysis was conducted to characterize the relationships.

For the analyses of seasonal host-parasitoid dynamics in South Africa, host fly density was estimated as the number of fly puparia per fruit. Because wild fruit is smaller than cultivated fruit, each wild fruit supports fewer flies, commonly one fly larvae per fruit^46^; therefore, the fly density per fruit approximately matches the percentage of infested fruit (i.e., fruit infestation rate). Data were pooled from different sites to estimate monthly mean host density or fruit infestation rate, total apparent parasitism by all braconid parasitoids, parasitoid diversity, and the relative abundance of each braconid parasitoids on unripe and ripe fruit, respectively. Linear mixed models were used to analyze the effects of fruit maturity (unripe vs. ripe) and seasonal climate (both were fixed effects) as well as year (random effect) on monthly host density and total parasitism. Host density data were square transformed while parasitism data were logit transformed as needed to meet normality and error variance for homoscedasticity. Monthly mean temperature was used to represent a seasonal climate variable as precipitation was considered similar within the surveyed areas and other temperature parameters (e.g., maximum or minimum temperature) are highly correlated with the mean temperature. The temperature data were obtained from Weather Information (https://us.worldweatheronline.com/) from the closest cities (Stellenbosch, Paarl, Citrusdal, Cape Town, Bonnievale or Wellington) of the sampled sites. Generalized linear models (GLM) were applied to analyze the effects of (1) fruit maturity, mean monthly temperature and host density on diversity, and (2) fruit maturity, mean monthly temperature, host density, and incidence of other parasitoids on the relative abundance of two major parasitoids (*P. lounsburyi* and *U. africanus*). For GLM analyses, fruit maturity was coded categorically as 1 and 2 for unripe and ripe fruit, respectively, and parasitoid species incidence was coded as 1 (present) and 0 (absent). Percentage data were modelled with binomial distribution and a logit link function while the diversity data was modeled with Poisson distribution and a log link function. Statistical analyses were performed using JMP Pro ver13 (SAS 2013, Cary, NC).

## Supplementary Information

Supplementary Information

## Data Availability

Data available from the Dryad Digital Repository (to be added).

## References

[1] Wolfe LM (2002). Why alien invaders succeed: Support for the escape-from-enemy hypothesis. Am. Nat..

[2] Facon B (2006). A general eco-evolutionary framework for understanding bioinvasions. Trends Ecol. Evol..

[3] Van Driesche RG (2010). Classical biological control for the protection of natural ecosystems. Biol. Control.

[4] Hajek AE (2016). Exotic biological control agents: A solution or contribution to arthropod invasions?. Biol. Invasions.

[5] Schwarzlander M, Hinz HL, Winston RL, Day MD (2018). Biological control of weeds: An analysis of introductions, rates of establishment and estimates of success, worldwide. Biocontrol.

[6] Naranjo SE, Ellsworth PC, Frisvold GB (2015). Economic value of biological control in integrated pest management of managed plant systems. Annu. Rev. Entomol..

[7] Hoddle MS, Lake EC, Minteer CR, Daane KM, Mason PG, Dennis N (2021). Biological Control: A Global Initiative.

[8] Heimpel GE, Cock MJW (2018). Shifting paradigms in the history of classical biological control. Biocontrol.

[9] Hoelmer KA, Kirk AA (2005). Selecting arthropod biological control agents against arthropod pests: Can the science be improved to decrease the risk of releasing ineffective agents?. Biol. Control.

[10] Wang XG, Johnson MW, Daane KM, Yokoyama VY (2009). Larger olive fruit size reduces the efficiency of *Psyttalia concolor*, as a parasitoid of the olive fruit fly. Biol. Control.

[11] Wang X-G, Levy K, Son Y, Johnson MW, Daane KM (2012). Comparison of the thermal performance between a population of the olive fruit fly and its co-adapted parasitoids. Biol. Control.

[12] Wharton RA, Robinson AS, Hooper G (1989). Fruit flies: Their Biology, Natural Enemies and Control.

[13] Purcell MF (1998). Contribution of biological control to integrated pest management of tephritid fruit flies in the tropics and subtropics. Integr. Pest Manag. Rev..

[14] Ovruski SM, Aluja M, Sivinski J, Wharton RA (2000). Hymenopteran parasitoids on fruit-infesting Tephritidae (Diptera) in Latin America and the southern United States: Diversity, distribution, taxonomic status and their use in fruit fly biological control. Integr. Pest Manag. Rev..

[15] Mohamed SA, Ramadan MM, Ekesi S, Ekesi S, Mohamed S, De Meyer M (2006). Fruit Fly Research and Development in Africa—Towards a Sustainable Management Strategy to Improve Horticulture.

[16] Garcia FRM, Ovruski SM, Suarez L, Cancino J, Liburd OE (2020). Biological control of tephritid fruit flies in the Americas and Hawaii: A review of the use of parasitoids and predators. Insects.

[17] Wharton, R. A. & Yoder, M. J. Wharton RA, Yoder MJ. 2017. Parasitoids of fruit-infesting tephritidae. http://paroffit.org. Accessed on November 15, 2020. (2017).

[18] Daane KM, Johnson MW (2010). Olive fruit fly: Managing an ancient pest in modern times. Annu. Rev. Entomol..

[19] Tzanakakis ME (2003). Seasonal development and dormancy of insects and mites feeding on olive: A review. Neth. J. Zool..

[20] Green PS (2002). A revision of *Olea* L. (Oleaceae). Kew Bull..

[21] Bon MC (2016). Populations of *Bactrocera oleae* (Diptera: Tephritidae) and its parasitoids in Himalayan Asia. Ann. Entomol. Soc. Am..

[22] Zygouridis NE, Augustinos AA, Zalom FG, Mathiopoulos KD (2009). Analysis of olive fly invasion in California based on microsatellite markers. Heredity.

[23] Augustinos AA (2005). Microsatellite analysis of olive fly populations in the Mediterranean indicates a westward expansion of the species. Genetica.

[24] Nardi F (2010). Domestication of olive fly through a multi-regional host shift to cultivated olives: Comparative dating using complete mitochondrial genomes. Mol. Phylogenet. Evol..

[25] Neuenschwander P, Bigler F, Delucchi V, Michelakis SE (1983). Natural enemies of preimaginal stages of *Dacus oleae* Gmel. (Dipt., Tephritidae) in Western Crete. I. Bionomics and phenologies. Boll Lab. Entomol Agrar Filippo Silvestri.

[26] Boccaccio L, Petacchi R (2009). Landscape effects on the complex of *Bactrocera oleae* parasitoids and implications for conservation biological control. Biocontrol.

[27] Borowiec N (2012). Diversity and geographic distribution of the indigenous and exotic parasitoids of the olive fruit fly, Bactrocera oleae (Diptera: Tephritidae) Southern France. IOBC/WPRS Bull..

[28] Al Khatib F (2014). An integrative approach to species discrimination in the Eupelmus urozonus complex (Hymenoptera, Eupelmidae), with the description of 11 new species from the Western Palaearctic. Syst. Entomol..

[29] Kapaun T, Nadel H, Headrick D, Vredevoe L (2010). Biology and parasitism rates of *Pteromalus* nr. *myopitae* (Hymenoptera: Pteromalidae), a newly discovered parasitoid of olive fruit fly *Bactrocera oleae* (Diptera: Tephritidae) in coastal California. Biol. Control.

[30] Silvestri F (1914). Report on an expedition to Africa in search of natural enemies of fruit flies (Trupaneidae) with descriptions, observations and biological notes. Hawaii Board Agric. For. Div. Entomol. Bull..

[31] Hoelmer KA, Kirk AA, Pickett CH, Daane KM, Johnson MW (2011). Prospects for improving biological control of olive fruit fly, *Bactrocera oleae* (Diptera: Tephritidae), with introduced parasitoids (Hymenoptera). Biocontrol Sci. Technol..

[32] Greathead DJ, Greathead AH (1992). Biological control of insect pests by insect parasitoids and predators: The BIOCAT database. Biocontrol News Inf..

[33] Neuenschwander P (1982). Searching parasitoids of Dacus oleae (Gmel) (Dipt., Tephritidae) in South Africa. J. Appl. Entomol..

[34] Loni A (1997). Developmental rate of *Opius concolor* (Hym.: Braconidae) at various constant temperatures. Entomophaga.

[35] Miranda MA, Miquel M, Terrassa J, Melis N, Monerris M (2008). Parasitism of *Bactrocera oleae* (Diptera, Tephritidae) by *Psyttalia concolor* (Hymenoptera, Braconidae) in the Balearic Islands (Spain). J. Appl. Entomol..

[36] Muller FA, Dias NP, Gottschalk MS, Garcia FRM, Nava DE (2019). Potential distribution of *Bactrocera oleae* and the parasitoids *Fopius arisanus* and *Psyttalia concolor*, aiming at classical biological control. Biol. Control.

[37] Chardonnet F, Blanchet A, Hurtrel B, Marini F, Smith L (2019). Mass-rearing optimization of the parasitoid *Psyttalia lounsburyi* for biological control of the olive fruit fly. J. Appl. Entomol..

[38] La-Spina M (2018). Effect of exposure time on mass-rearing production of the olive fruit fly parasitoid, *Psyttalia lounsburyi* (Hymenoptera: Braconidae). J. Appl. Entomol..

[39] Malausa JC (2010). Introductions of the African parasitoid Psyttalia lounsburyi in South of France for classical biological control of Bactrocera oleae. IOBC/WPRS Bull..

[40] Daane KM (2015). Classic biological control of olive fruit fly in California, USA: Release and recovery of introduced parasitoids. Biocontrol.

[41] Wharton RA, Gilstrap F (1983). Key to and status of opiine braconid (Hymenoptera) parasitoids used in biological control of *Ceratitis* and *Dacus* s.l. (Diptera: Tephritidae). Ann. Entomol. Soc. Am..

[42] Sime KR (2007). *Psyttalia ponerophaga* (Hymenoptera: Braconidae) as a potential biological control agent of olive fruit fly *Bactrocera oleae* (Diptera: Tephritidae) in California. Bull. Entomol. Res..

[43] Sime KR (2006). The biology of *Bracon celer* as a parasitoid of the olive fruit fly. Biocontrol.

[44] Sime KR (2006). *Diachasmimorpha longicaudata* and *D. kraussii* (Hymenoptera: Braconidae), potential parasitoids of the olive fruit fly. Biocontrol Sci. Technol..

[45] Sime KR, Daane KM, Messing RH, Johnson MW (2006). Comparison of two laboratory cultures of *Psyttalia concolor* (Hymenoptera: Braconidae), as a parasitoid of the olive fruit fly. Biol. Control.

[46] Mkize N, Hoelmer KA, Villet MH (2008). A survey of fruit-feeding insects and their parasitoids occurring on wild olives, *Olea europaea* ssp *cuspidata*, in the Eastern Cape of South Africa. Biocontrol Sci. Technol..

[47] Sime KR, Daane KM, Wang X-G, Johnson MW, Messing RH (2008). Evaluation of *Fopius arisanus* as a biological control agent for the olive fruit fly in California. Agric. For. Entomol..

[48] Wang XG, Messing RH (2004). Potential interactions between pupal and egg- or larval-pupal parasitoids of tephritid fruit flies. Environ. Entomol..

[49] Wang XG, Messing RH (2004). The ectoparasitic pupal parasitoid, *Pachycrepoideus vindemmiae* (Hymenoptera: Pteromalidae), attacks other primary tephritid fruit fly parasitoids: Host expansion and potential non-target impact. Biol. Control.

[50] Wang X-G, Johnson MW, Yokoyama VY, Pickett CH, Daane KM (2011). Comparative evaluation of two olive fruit fly parasitoids under varying abiotic conditions. Biocontrol.

[51] Daane KM (2011). Biological control of the olive fruit fly in California. Calif. Agric..

[52] Wang XG (2009). Crop domestication relaxes both top-down and bottom-up effects on a specialist herbivore. Basic Appl. Ecol..

[53] Nadel H, Daane KM, Hoelmer KA, Pickett CH, Johnson MW (2009). Non-target host risk assessment of the idiobiont parasitoid, *Bracon celer* (Hymenoptera: Braconidae), for biological control of olive fruit fly in California. Biocontrol Sci. Technol..

[54] Wharton RA (2000). Parasitoids of medfly, *Ceratitis capitata*, and related tephritids in Kenyan coffee: A predominantly koinobiont assemblage. Bull. Entomol. Res..

[55] Kimani-Njogu SW, Trostle MK, Wharton RA, Woolley JB, Raspi A (2001). Biosystematics of the *Psyttalia concolor* species complex (Hymenoptera: Braconidae: Opiinae): the identity of populations attacking *Ceratitis capitata* (Diptera: Tephritidae) in coffee in Kenya. Biol. Control.

[56] Rugman-Jones PF, Wharton R, van Noort T, Stouthamer R (2009). Molecular differentiation of the *Psyttalia concolor* (Szépligeti) species complex (Hymenoptera: Braconidae) associated with olive fly, *Bactrocera oleae* (Rossi) (Diptera: Tephritidae), Africa. Biol. Control.

[57] Billah MK (2008). Cross mating studies among five fruit fly parasitoid populations: Potential biological control implications for tephritid pests. Biocontrol.

[58] Narayanan ES, Chawla SS (1962). Parasites of fruit fly pests of the world. Beitrage zur Entomologie.

[59] Neuenschwander P (1982). Searching parasitoids of *Dacus oleae* in South Africa. Zeitschrift fur Angewandte Entomologie.

[60] Daane KM (2008). *Psyttalia lounsburyi* (Hymenoptera: Braconidae), potential biological control agent for the olive fruit fly in California. Biol. Control.

[61] Benelli G (2013). Behavioral and electrophysiological responses of the parasitic wasp *Psyttalia concolor* (Szepligeti) (Hymenoptera: Braconidae) to *Ceratitis capitata*-induced fruit volatiles. Biol. Control.

[62] Raspi A, Loni A (1994). Alcune note sull’allevamento massale di Opius concolor Szépligeti (Hym.: Braconidae) e su recnti tentative d’introduzione della specie in Toscana e Liguria. Frustula Entomol..

[63] Johnson MW (2011). High temperature impacts olive fruit fly population dynamics in California’s Central Valley. Calif. Agric..

[64] Yokoyama VY (2012). Performance of *Psyttalia humilis* (Hymenoptera: Braconidae) reared from irradiated host on olive fruit fly (Diptera: Tephritidae) in California. Environ. Entomol..

[65] Yokoyama VY (2010). Response of Psyttalia cf. concolor to olive fruit fly (Diptera: Tephritidae), high temperature, food, and bait sprays in California. Environ. Entomol..

[66] Yokoyama VY (2010). Field performance and fitness of an olive fruit fly parasitoid, *Psyttalia humilis* (Hymenoptera: Braconidae), mass reared on irradiated Medfly. Biol. Control.

[67] Wang XG (2013). Overwintering survival of olive fruit Fly (Diptera: Tephritidae) and two introduced parasitoids in California. Environ. Entomol..

[68] Daane KM, Wang XG, Johnson MW, Cooper ML (2013). Low temperature storage effects on two olive fruit fly parasitoids. Biocontrol.

[69] R Core Team. R: A Language and Environment for Statistical Computing. R Foundation for Statistical Computing, Vienna, Austria. (accessed 20 Dec 2020); https://www.r-project.org/index.htm (2020).

