# Supplementary material for: Exploration for olive fruit fly parasitoids across Africa reveals regional distributions and dominance of closely associated parasitoids
Source: Sci Rep. 2021 Mar 17;11:6182. doi: 10.1038/s41598-021-85253-y (PMC7971055; doi:10.1038/s41598-021-85253-y)
Supplement: Supplementary file 1 — Supplementary Information [file 41598_2021_85253_MOESM1_ESM.docx]

**Exploration for olive fruit fly parasitoids across Africa reveals regional distributions and dominance of closely associated parasitoids**

Xingeng Wang^1,2*^, Vaughn M. Walton^1,3^, Kim A. Hoelmer^2,4^, Charles H. Pickett^5^, Arnaud Blanchet^4^, Robert K. Straser^1,6^, Alan A. Kirk^4^ & Kent M. Daane^1*^

^1^ Department of Environmental Science, Policy and Management, University of California, Berkeley, CA 94720, USA. ^2^ Present address: USDA-ARS Beneficial Insects Introduction Research Unit, Newark, DE 19713, USA. ^3^ Present address: Department of Horticulture, Oregon State University, Corvallis, OR 97331, USA. ^4^ USDA-ARS, European Biological Control Laboratory, Montferrier, France. ^5^ California Department of Food and Agriculture, Sacramento, CA 95832, USA. ^6^ Present address: Department of Entomology, University of California, Riverside, Riverside, CA 92521, USA.

*Corresponding authors, emails:[kmdaane@berkeley.edu](mailto:kmdaane@berkeley.edu); [xingeng.wang@usda.gov](mailto:xingeng.wang@usda.gov)

African collections for the olive fruit fly, *Bactrocera oleae*, and its parasitoids were conducted from 2000-2011 in seven regions: parts of Kenya, Namibia, South Africa, the Canary Islands, Tunisia, Morocco and Réunion Island. Supplemental Figure 1 provides closer detailed locations for collections of wild olives infested by *Bactrocera* spp. in these seven sampled regions. Table S1 provides a comparison of sex ratio (% females) of each parasitoid species collected from different regions. Supplemental material S2-S10 provides the diagnostic plots for all analyses of ANOVAs, Mixed models and GLMs and statistical outputs; all statistical figures and analyses were performed using JMP Pro ver13 (SAS 2013, Cary, NC).

Supplemental Figure 1 (Fig. S1). Detailed sampling locations for collections of wild olives infested by *Bactrocera* spp. in (A) Reunion Islands, (B) Kenya, (C) Canary Islands, (D) Morocco, (E) Namibia, (F) South Africa and (G) Tunisia. Each red dot represents a different location based on GPS coordinates. The map was created in R (version 3.6.3, www.r-project.org) using “get_map” wrapper from “ggmap” package (version 3.0.0, <https://cran.r-project.org/web/> packages/ggmap/ggmap.pdf), which queries with Google Maps server to produce static maps.


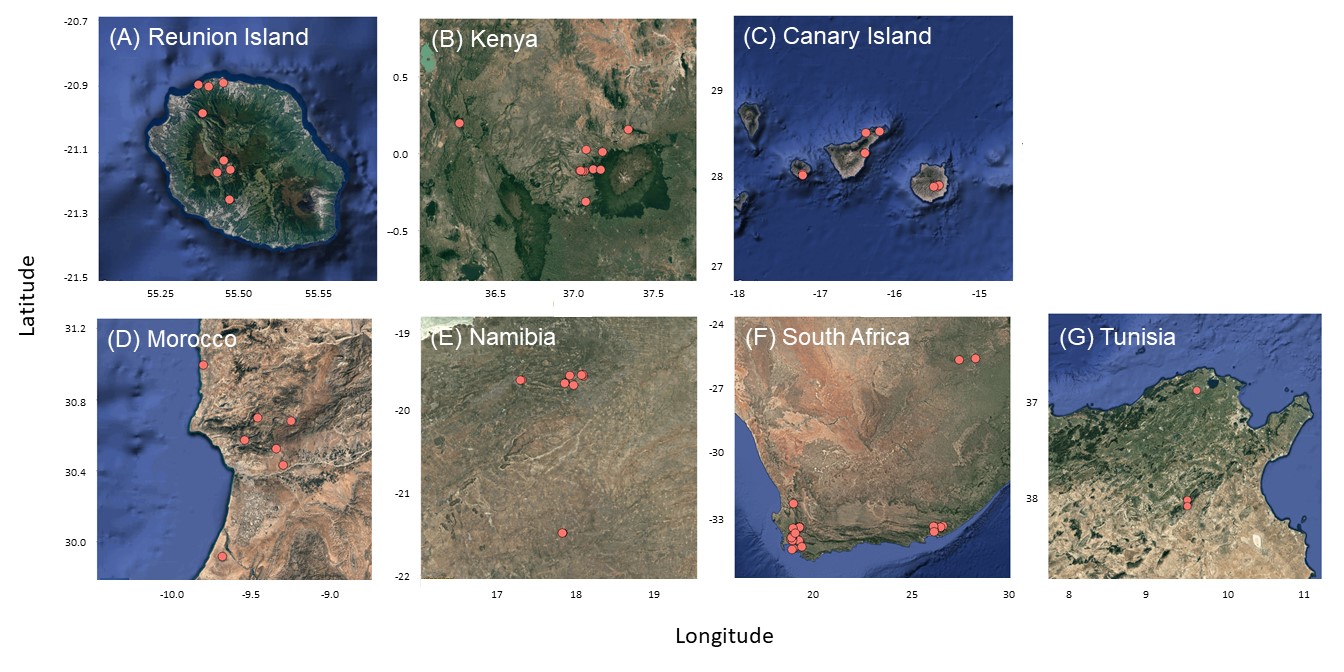


Table S1. Comparison of sex ratio (% females) of each parasitoid species collected from different regions

| Parasitoid species* | Regions | Mean ± SE | *F* | *P* |
| --- | --- | --- | --- | --- |
| *B. celer* | Namibia | 27.3 ± 6.2 | *F*_1,33_ = 1.135 | 0.295 |
|  | South Africa | 22.2 ± 7.5 |  |  |
|  |  |  |  |  |
| *P. concolor* | Canary Islands | 88.0 ± 12.0 | *F*_2,8_ = 0.979 | 0.416 |
|  | Morocco | 50.8 ± 20.6 |  |  |
|  | Tunisia | 51.1 ± 15.4 |  |  |
|  |  |  |  |  |
| *P. humilis* | Namibia | 62.8 ± 6.6 | *F*_1,26_ = 0.403 | 0.531 |
|  | South Africa | 70.0 ± 13.1 |  |  |
|  |  |  |  |  |
| *P. lounsburyi* | Kenya | 45.4 ± 3.0 | *F*_2,39_ = 2.890 | 0.068 |
|  | Namibia | 73.8 ± 9.9 |  |  |
|  | South Africa | 45.8 ± 7.5 |  |  |
|  |  |  |  |  |
| *U. africanus* | Kenya | 56.8 ± 4.7 | *F*_2,48_ = 0.306 | 0.738 |
|  | Namibia | 61.9 ± 7.1 |  |  |
|  | South Africa | 62.0 ± 6.3 |  |  |

**D.* sp. nr. *fulllawayi* was collected only from Reunion.

S2 – One-way ANONA comparing regional differences in total parasitism. Data were logit transformed as needed to meet for normality and error variance for homoscedasticity.


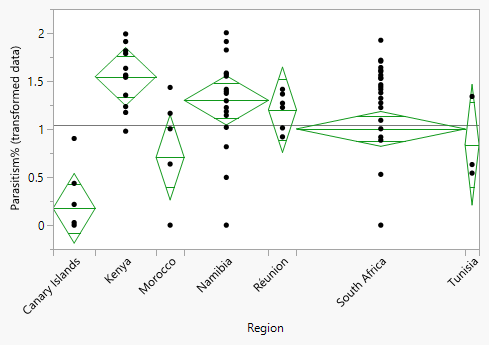


| Source | DF | Sum of Squares | Mean Square | F Ratio | Prob > F |
| --- | --- | --- | --- | --- | --- |
| Region | 6 | 12.275 | 2.045 | 6.837 | <.0001* |
| Error | 84 | 25.135 | 0.299 |  |  |
| C. Total | 90 | 37.410 |  |  |  |

S3 – One-way ANONA comparing regional differences in the diversity of parasitoid species.


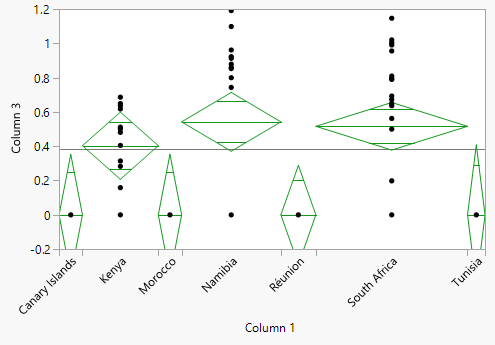


| Source | DF | Sum of Squares | Mean Square | F Ratio | Prob > F |
| --- | --- | --- | --- | --- | --- |
| Region | 6 | 3.396 | 0.566 | 4.509 | 0.0007* |
| Error | 66 | 8.284 | 0.125 |  |  |
| C. Total | 72 | 11.680 |  |  |  |

S4 – One-way ANONA comparing the difference in sex ratio among different parasitoid species. Data were logit transformed as needed to meet for normality and error variance for homoscedasticity


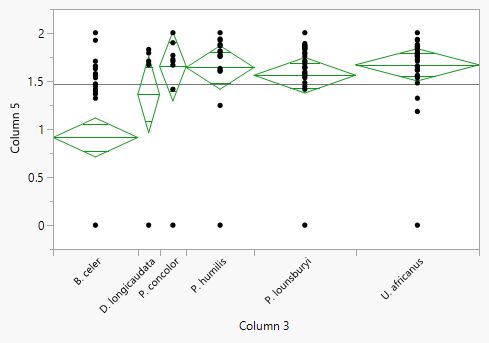


| Source | DF | Sum of Squares | Mean Square | F Ratio | Prob > F |
| --- | --- | --- | --- | --- | --- |
| Parasitoid species | 5 | 14.561 | 2.912 | 7.899 | <.0001* |
| Error | 170 | 62.670 | 0.368 |  |  |
| C. Total | 175 | 77.231 |  |  |  |

S5 – One-way ANONA comparing the difference in pulp thickness between unripe and ripe olive fruit.


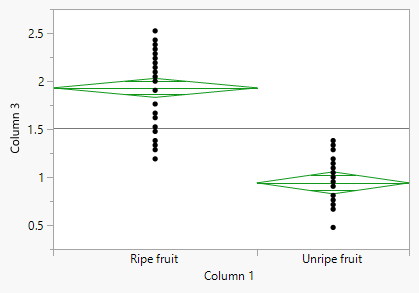


| Source | DF | Sum of Squares | Mean Square | F Ratio | Prob > F |
| --- | --- | --- | --- | --- | --- |
| Fruit maturity | 1 | 17.972 | 17.972 | 169.641 | <.0001* |
| Error | 73 | 7.734 | 0.105 |  |  |
| C. Total | 74 | 25.706 |  |  |  |

S6 – Linear Mixed Model analyzing the effects of fruit maturity (unripe vs. ripe), seasonal climate (both were fixed effects) and their interaction as well as year (random effect) on monthly % fruit infested. Data were square transferred as needed to meet for normality and error variance for homoscedasticity.


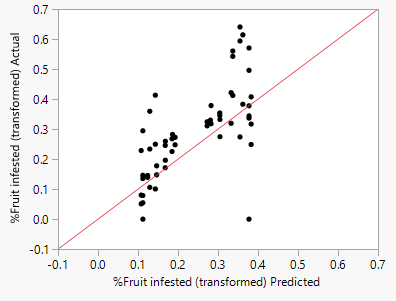


| Source | Nparm | DFNum | DFDen | F Ratio | Prob > F |
| --- | --- | --- | --- | --- | --- |
| Fruit maturity | 1 | 1 | 48.7 | 60.135 | <.0001* |
| Mean temperature | 1 | 1 | 50.2 | 0.100 | 0.752 |
| Fruit maturity*Mean temperature | 1 | 1 | 48.7 | 6.887 | 0.012* |

S7 – Linear Mixed Models analyzing the effects of fruit maturity (unripe vs. ripe) and seasonal climate (both were fixed effects) and their interaction as well as year (random effect) on monthly % parasitism. Parasitism data were logit transformed as needed to meet for normality and error variance for homoscedasticity.


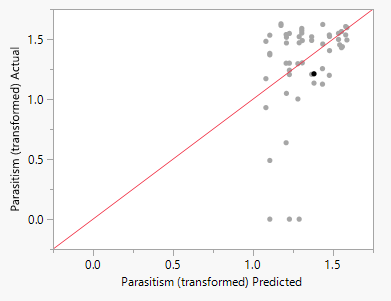


| Source | Nparm | DFNum | DFDen | F Ratio | Prob > F |
| --- | --- | --- | --- | --- | --- |
| Fruit maturity | 1 | 1 | 48.8 | 0.554 | 0.460 |
| Mean temperature | 1 | 1 | 40.6 | 6.549 | 0.014* |
| Fruit maturity*Mean temperature | 1 | 1 | 48.8 | 0.153 | 0.697 |

S8 – Generalized linear models (GLM) analyzing the effects of fruit maturity (coded categorically as 1 and 2 for unripe and ripe fruit, respectively), mean monthly temperature and host density on the diversity of parasitoids.


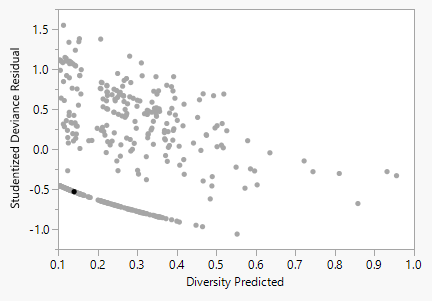


| Term | Estimate | Std Error | L-R ChiSquare | Prob>ChiSq | Lower CL | Upper CL |
| --- | --- | --- | --- | --- | --- | --- |
| Intercept | -3.168 | 0.475 | 55.310 | <.0001* | -4.133 | -2.266 |
| Fruit maturity | 0.662 | 0.223 | 9.449 | 0.002* | 0.235 | 1.114 |
| Mean temperature | 0.031 | 0.023 | 1.844 | 0.174 | -0.014 | 0.077 |
| Host density | 1.211 | 0.430 | 7.019 | 0.008* | 0.329 | 2.022 |

S9 – Generalized linear models (GLM) analyzing the effects of fruit maturity (coded categorically as 1 and 2 for unripe and ripe fruit, respectively), mean monthly temperature, host density as well as incidence of other parasitoid species (code as 1= presence or 0=absence) on the relative abundance of the major parasitoid *U. africanus*.


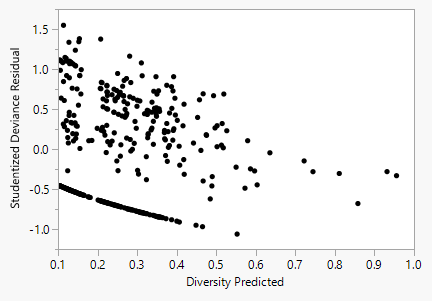


| Term | Estimate | Std Error | L-R ChiSquare | Prob>ChiSq | Lower CL | Upper CL |
| --- | --- | --- | --- | --- | --- | --- |
| Intercept | 4.293 | 0.668 | 50.923 | <.0001* | 3.029 | 5.655 |
| Fruit maturity | -0.644 | 0.301 | 4.707 | 0.030* | -1.243 | -0.061 |
| Mean temperature | -0.040 | 0.033 | 1.499 | 0.220 | -0.104 | 0.024 |
| Host density | 2.175 | 0.809 | 7.916 | 0.005* | 0.641 | 3.831 |
| Presence of *P. lounsburyi* | -2.627 | 0.315 | 90.086 | <.0001* | -3.274 | -2.033 |
| Presence of *P. humilis* | -1.644 | 0.403 | 16.879 | <.0001* | -2.448 | -0.861 |
| Presence of *B. celer* | -1.973 | 0.529 | 15.115 | <.0001* | -3.051 | -0.964 |

S10 – Generalized linear models (GLM) analyzing the effects of (1) fruit maturity (coded categorically as 1 and 2 for unripe and ripe fruit), mean monthly temperature, host density as well as incidence of other parasitoid species (code as 1= presence or 0=absence) on the relative abundance of the major parasitoid *P. lounsburyi*.


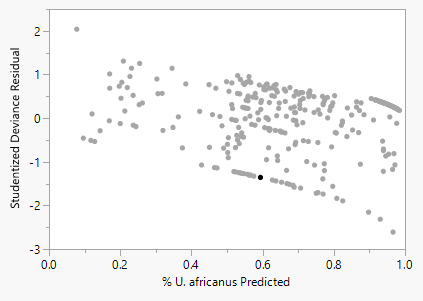


| Term | Estimate | Std Error | L-R ChiSquare | Prob>ChiSq | Lower CL | Upper CL |
| --- | --- | --- | --- | --- | --- | --- |
| Intercept | -1.559 | 0.767 | 4.179 | 0.041* | -3.085 | -0.064 |
| Fruit maturity | 0.845 | 0.316 | 7.523 | 0.006* | 0.238 | 1.483 |
| Mean temperature | 0.075 | 0.034 | 4.778 | 0.029* | 0.008 | 0.143 |
| Host density | 1.755 | 0.739 | 5.398 | 0.020* | 0.283 | 3.200 |
| Presence of *U. africanus* | -2.958 | 0.433 | 50.484 | <.0001* | -3.843 | -2.133 |
| Presence of *P. humilis* | -1.268 | 0.543 | 6.681 | 0.009* | -2.442 | -0.285 |
| Presence of *B. celer* | -0.183 | 0.566 | 0.105 | 0.745 | -1.351 | 0.886 |
